# Genome-Wide Identification of Alternatively Spliced mRNA Targets of Specific RNA-Binding Proteins

**DOI:** 10.1371/journal.pone.0000520

**Published:** 2007-06-13

**Authors:** Mark D. Robida, Andrew Rahn, Ravinder Singh

**Affiliations:** Department of Molecular, Cellular and Developmental Biology, University of Colorado at Boulder, Boulder, Colorado, United States of America; Centre de Regulació Genòmica, Spain

## Abstract

**Background:**

Alternative splicing plays an important role in generating molecular and functional diversity in multi-cellular organisms. RNA binding proteins play crucial roles in modulating splice site choice. The majority of known binding sites for regulatory proteins are short, degenerate consensus sequences that occur frequently throughout the genome. This poses an important challenge to distinguish between functionally relevant sequences and a vast array of those occurring by chance.

**Methodology/Principal Findings:**

Here we have used a computational approach that combines a series of biological constraints to identify uridine-rich sequence motifs that are present within relevant biological contexts and thus are potential targets of the *Drosophila* master sex-switch protein Sex-lethal (SXL). This strategy led to the identification of one novel target. Moreover, our systematic analysis provides a starting point for the molecular and functional characterization of an additional target, which is dependent on SXL activity, either directly or indirectly, for regulation in a germline-specific manner.

**Conclusions/Significance:**

This approach has successfully identified previously known, new, and potential SXL targets. Our analysis suggests that only a subset of potential SXL sites are regulated by SXL. Finally, this approach should be directly relevant to the large majority of splicing regulatory proteins for which bonafide targets are unknown.

## Introduction

Intervening sequences called introns interrupt the majority of genes in multi-cellular organisms. Spliceosomal introns are rare (<4%) in the budding yeast but present in the majority (85–94%) of genes in metazoans. Introns are removed and the coding regions (exons) joined together via a process known as pre-mRNA splicing before an mRNA can be translated. The 5′ and 3′ splice sites, the branchpoint, and the polypyrimidine tract (Py-tract) are important splicing signals in metazoans. Five small nuclear ribonucleoproteins (U1, U2, U4, U5, and U6 snRNPs), along with several additional factors, recognize these signals and assemble onto the pre-mRNA to form a large complex called the spliceosome. Spliceosome assembly occurs in several distinct steps, involving RNA-RNA, protein-protein, and RNA-protein interactions, leading to two catalytic reactions [1], [2].

Alternative splicing generates multiple mRNA and/or protein isoforms from a single gene through the use of alternative 5′ splice sites, 3′ splice sites, exons, and/or introns. Several genes are known to encode >1,000 alternatively spliced mRNA isoforms. For example, the *Drosophila* homolog of the human Down Syndrome Cell Adhesion Molecule (DSCAM) gene potentially encodes three times as many alternatively spliced transcripts (∼38,000) as the total number of predicted genes (∼13,600) in the fruitfly [3], [4]. Thus, alternative splicing, among several processes [5], provides a mechanism to generate enormous molecular diversity from a single gene, and provides a rich source of functional diversity in multi-cellular eukaryotes [6], [7]. Alternative splicing plays an important role in numerous cellular and developmental processes such as cell growth and differentiation, cell signaling, programmed cell death, and nonsense-mediated decay [8], [9].

The best-studied example of a developmental process controlled by alternative splicing is the *Drosophila melanogaster* somatic sex-determination pathway. It involves a hierarchy of alternative splicing events in which the key sex determining genes (*Sex-lethal* (*Sxl*), *transformer* (*tra*), *double-sex* (*dsx*), and *male specific-lethal 2* (*msl2*) are spliced differently in male (XY) and female (XX) flies. The master sex-switch protein, SXL, is an RNA-binding protein that is absent in male flies and present in females [10]. It affects the splicing of three known pre-mRNAs by binding to uridine-rich sequences or polypyrimidine-tracts (Py-tracts) that are present adjacent to splice sites, leading to exon skipping in *Sxl*, 3′ splice site switching in *tra*, and intron retention in *msl2* [6], [11]. In female somatic cells, SXL mediates sexual differentiation and courtship behavior by allowing synthesis of the TRA protein, and allows proper dosage compensation by preventing synthesis of the MSL2 protein [10], [11]. In addition to its role in alternative splicing, SXL also represses translation by binding to uridine-rich sequences in the untranslated regions (UTRs) of the *Sxl* and *msl2* mRNAs [11]. Furthermore, SXL also controls female germline development [10]. Absence of SXL in the female germline causes mitotic and meiotic defects, resulting in ovarian tumors or multicellular cysts of small undifferentiated cells [12], [13], [14] and in defects in chromosome pairing and meiotic recombination [15].

Several independent studies have suggested that additional targets of SXL exist. First, SXL associates with numerous loci on polytene X-chromosomes, presumably binding to nascent transcripts [16]. Second, SXL regulates the *fit* (*female-specific independent of tra*) gene in a *tra*-independent manner in the soma, although it is unlikely to be a direct target of SXL because of the lack of sex-specific mRNA isoforms and lack of SXL-binding sites [17]. Third, SXL controls dosage compensation of some *msl2*-independent gene(s) that remains to be identified [18]. Fourth, although SXL has several important functions in the female germline, previous attempts to develop a genetic handle on its germline-specific targets have been unsuccessful [19]. Thus, additional targets of SXL, particularly in the female germline, have gone unrecognized, most likely because of subtle phenotypes, redundant functions, or limitations of a particular genetic screen.

Here we present a computational strategy that allowed identification of both new and potential SXL targets. This approach may be used to identify potential targets of other RNA binding proteins.

## Results

### Computational strategy for the identification of potential targets of SXL

Given that the *Drosophila* genome has been sequenced [3] and that the SXL-binding site has been well characterized [20], [21], [22], [23], we searched the entire *Drosophila* genome using a weight matrix corresponding to the SXL-binding site. Unlike string matching, this approach provides a quantitative rather than a merely qualitative description of a binding site by assigning weights to the four nucleotides at each sequence position. We aligned the SXL-binding sites (UUUUGUU(G/U)U(G/U)UUU(G/U)UU) from sequences selected by SELEX from a random RNA library [20] and converted this alignment matrix into a weight matrix of log-likelihood scores (Supplementary Table S1), as described [24]. We searched each of the overlapping 16-nucleotide strings in the *Drosophila* genome and calculated the total score for each string based on the weight matrix. If the score was above a user defined cut-off value (5.1 was used here to obtain only high-affinity binding sites), the genomic location of the binding site was saved. However, if the score was below the cut-off, the search engine advanced to the next position (for additional details see materials and methods).

This score was carefully chosen to capture known high affinity, long SXL sites such as those adjacent to regulated splice sites of *tra*, *Sxl*, and *msl2* transcripts, but ignore the majority of the short Py-tracts, including those associated with 3′ splice sites. We empirically determined how far apart the hits were in the genome sequence. For example, when the hits were on average 20,000 basepairs apart, we expected approximately 12,600 binding sites. The cut-off (5.1) used here ignored most of the uridine-tracts such as those present near 3′ splice sites. We were aware that it eliminated multiple copies of clustered, short Py-tracts, which might be potentially regulated, because the number of hits became unmanageable. For the matrix for the SXL-binding site, a maximum possible score is 7.88 (Table S1).

**Figure 1:**
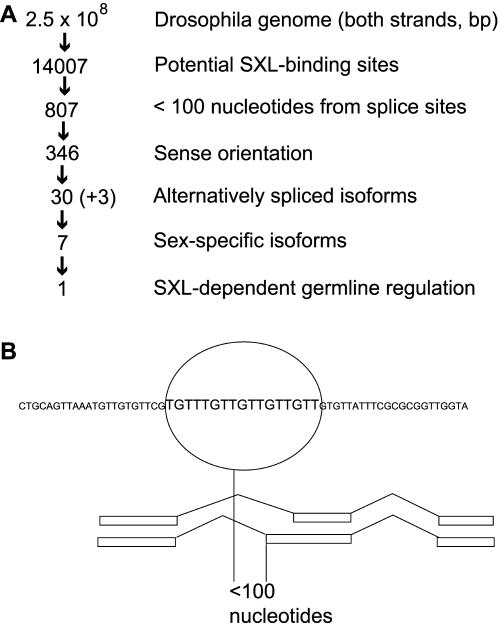
Computational search identifies potential targets of SXL. (A) Step-wise rationale for the identification of biologically relevant SXL targets. The number of potential candidates with SXL binding sites remaining after each step is indicated. (+3) represents that the three known targets of SXL (*Sxl, tra, msl2*) were also identified. (B) Schematics of how the search program works. Overlapping nucleotide windows are scanned for sequences that match the consensus-binding site. When a binding site is identified, the search program determines whether it is within 100 nucleotides of a splice site, whether it is in the sense orientation, and whether there is evidence of alternative splicing for that site.

Our search of both strands of the genomic DNA yielded 14,007 matches for putative high affinity SXL-binding sites (Figure 1A). Given that there are approximately 13,600 predicted genes in *Drosophila* [3], the initial number of matches was too large for experimental analysis. Therefore, our *in silico* analysis included the following filters in a step-wise manner to reduce the number of candidates to an experimentally manageable size (Figure 1B). First, we determined if there was an expressed sequence tag (EST) within 3 kb on either side of the potential SXL-binding sites. This was intended to eliminate the matches that were in the intergenic region, which is particularly AT-biased [3]. Second, since SXL controls the splicing of its known targets, the remaining candidates were filtered on the basis of the proximity of potential binding sites to known splice sites. For the initial screen, we selected only those candidates (807) in which SXL-binding sites were located within 100 nucleotides of known 5′ or 3′ splice sites. The splice site locations were assigned based on comparison of EST sequences to the genomic sequence and on their match to the splice site consensus [25]. Third, we discarded the candidates that were not present on the sense strand of the relevant genes. This left us with 346 candidates. Fourth, since all known targets of SXL are regulated by alternative splicing, we determined whether there was evidence of alternative splicing for the potential candidates based on the database of about 86,000 ESTs. We determined if splice sites adjacent to potential SXL-binding sites were alternatively spliced by aligning EST sets for each of the 346 candidates, using the ClustalW multiple sequence alignment program. The total number of potential candidates that met these multiple criteria was 33 (30 new) (Figure 1A); this number was experimentally amenable. It should be emphasized that this list included all 3 previously known targets of SXL (*Sxl*, *tra*, and *msl2*), and that several candidates contained multiple SXL-binding sites. Thus, this strategy successfully identified all previously known SXL targets as well as potential new targets.

### Seven candidates show sex-specific mRNA isoforms in adult flies

**Figure 2:**
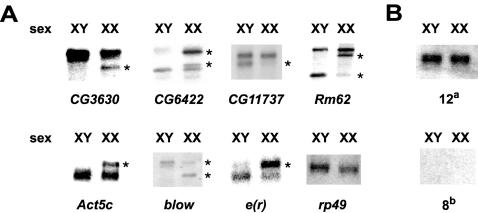
Screening for sex-specific isoforms. (A) Seven candidates show sex-specific mRNA isoforms by Northern analysis on poly(A)^+^ RNA from adult flies. Asterisks indicate sex-specific isoforms. XY and XX indicate chromosomal sex. (B) (Top) Several candidates (*bancal*, *Bap60*, *ImpL3*, *inx7*, *Moe*, *Rala*, *Ric*, *Top2*, *Frq2*, *Cyp28a5*, *RpL36*, and *Dhc16F*)^a^ were present at equal levels in both sexes. (Bottom) Several others (*fus*, *pUf68*, CG8370, *katanin-60*, *vap*, CG2967, CG5455, and *aralar1*)^b^ showed no hybridization in adult flies.

SXL is present in females and absent in males. Furthermore, all known targets of SXL are alternatively spliced in a sex-specific manner, generating sex-specific isoforms that differ in length and that can be identified using Northern analysis. Thus, it was anticipated that at least some of the additional candidates would also generate sex-specific isoforms. To pursue them, we obtained cDNA clones for each of the 30 new candidates and performed Northern analysis using poly(A)^+^ RNA from male or female adult flies. Twelve candidates showed expression but no sex-specific isoforms, and eight candidates showed no detectable signal on these RNA blots from adult flies (Figure 2). It remains possible that these candidates have similarly sized alternative exons, low abundance sex-specific transcripts, or sex-specific expression at other stages during development. These candidates were not pursued at this stage. Most importantly, seven candidates showed sex-specific mRNA isoforms (Figure 2, see asterisks), indicating that they might be potential SXL targets. CG3630 was found to have a longer non-sex-specific transcript and a shorter female-specific transcript. CG6422 had a shorter non-sex-specific transcript and a pair of longer female-specific transcripts. CG11737 had a longer non-sex-specific transcript and a shorter male-specific transcript. *Rm62* had a longer non-sex-specific transcript and two shorter sex-specific transcripts, one male-specific and the other female-specific. *Act5c* and *e(r)* both had a shorter non-sex-specific transcript and a longer female-specific transcript. Finally, *blow* had a longer non-sex-specific transcript and two shorter transcripts, one male-specific and another female-specific.

### SXL-binding site and sex-specific isoforms

**Figure 3:**
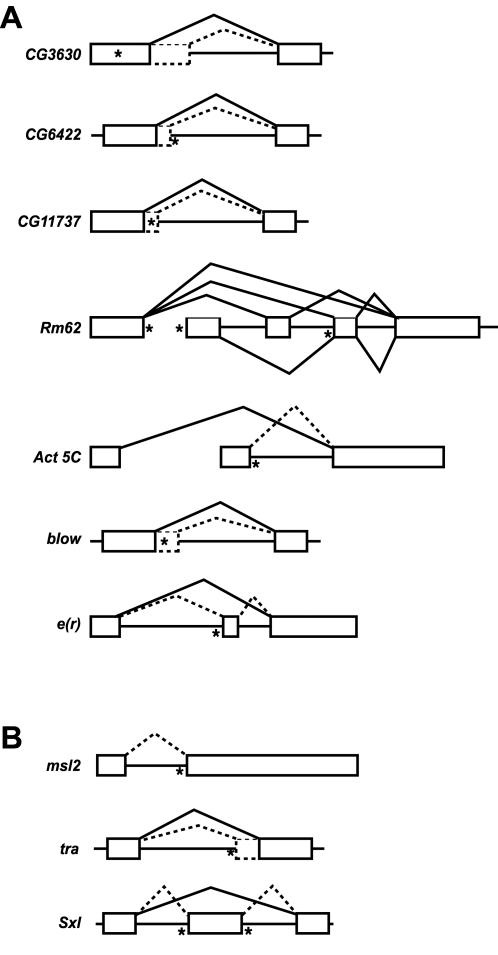
Schematics of the relevant portion of the potential SXL targets (A) and known SXL targets (B). Boxes, exons; horizontal lines, introns; solid and dotted lines, alternative splicing pathways; asterisks, potential SXL-binding sites. The SXL-binding sites identified are: CG3630 (UUUUUCUUGUUUUUUUU), CG6422 (UUUUUGUUUUUUUUUU), CG11737 (UUUUUGUUGUUUUUUUUUUU), *Rm62* (UUUUUUUUUUUUUUUUU, UUUGUUGUUUUUUUCUUUGUGUUUG, and UUUUUUUU), *Act5c* (GUGUUUUUUUUUUUUUUU), *blow* (UUUUUUUUUUUUUUUUUUUUUUUUUUUGUUU), and *e(r)* (UUUUUUUUUUGUCUUUUUUUUUUUU and UGUGUGUGUUUUUGUGUGUUUCAAUGUUUUUUUGUG).

Examination of the EST database revealed potential sources for the sex-specific differences and suggested how SXL might regulate these targets (Figure 3A). Four candidates (CG3630, CG6422, CG11737, and *blow*) showed evidence of alternative 5′ splice site choice adjacent to the SXL-binding site. The SXL-binding site in CG3630 was found within the exon upstream of the first alternative 5′ splice site, the SXL-binding site in CG6422 was found downstream of the second alternative 5′ splice site, and the SXL-binding sites in CG11737 and *blow* were found between alternative 5′ splice sites. These scenarios are reminiscent of the way in which SXL regulates 3′ splice site choice in its known target *tra* (Figure 3B) by binding to a site adjacent to the non-sex-specific 3′ splice site [26], [27]. Three candidates (*Rm62*, *Act5c*, and *e(r)*) showed evidence of alternative exon usage near SXL binding sites (Figure 3A). *Rm62* contained three identified SXL-binding sites adjacent to alternative exons. *Act5c* and *e(r)* had SXL-binding sites located adjacent to alternative exons. Since the difference in the size of the alternative *Act5c* exons alone is insufficient to account for the sex-specific isoforms we believe that the female-specific isoform most likely reflects cross-hybridization to different members of the highly conserved actin family [28]. The use of alternative exons in *Rm62* and *e(r)* is reminiscent of the regulation of the known target *Sxl* (Figure 3B), in which exon skipping is caused by SXL binding to sites flanking an alternative exon [29], [30]. As noted above, the new target *e(r)* was one of the candidates that contained multiple SXL-binding sites; one adjacent to an alternatively spliced exon and another downstream of an alternative polyadenylation site. Our molecular characterization of *e(r)* showed that both alternatively spliced and alternatively polyadenylated transcripts exist *in vivo* (data not shown). We found that the latter makes the primary contribution to sex-specific regulation, which occurs specifically in the female germline [31]. This candidate was pursued in significant detail because its regulation involved a novel mechanism. Our extensive molecular genetic analysis involving mutations in *Sxl* and the SXL-binding site and biochemical analysis using recombinant proteins showed that SXL-dependent regulation of *e(r)* provides a molecular mechanism for translational repression specifically within the female germline [31].

### Somatic versus germline expression of the remaining sex-specific transcripts

**Figure 4:**
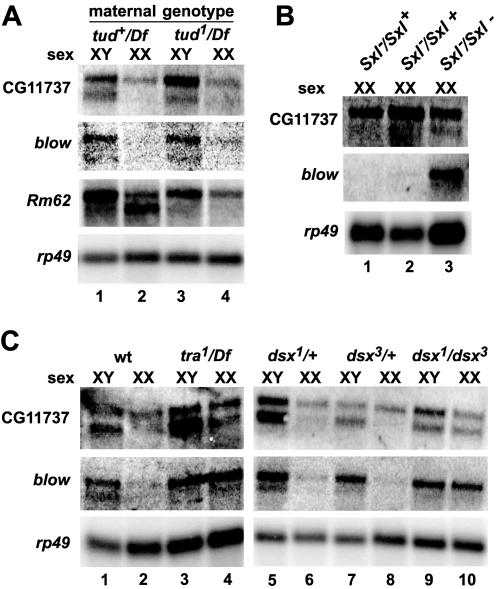
The sex-specific transcripts of CG11737 and *blow* are downstream of *dsx* in the soma. A. The sex-specific transcripts of CG11737 and *blow* are somatic in origin, and the sex-specific transcript of *Rm62* is restricted to the germline. CG11737 and blow show no changes in expression pattern in the progeny of *tud* mothers (lanes 3 and 4 versus 1 and 2), while the female-specific transcript of *Rm62* is lost (lane 4 versus lane 2). B. The sex-specific transcripts of CG11737 and *blow* are downstream of *Sxl* in the soma. Loss of *Sxl* in XX flies causes a switch to the male expression pattern (lane 3). C. The sex-specific transcripts of CG11737 and *blow* are downstream of *tra* and *dsx* in the soma. Loss of *tra* (lane 4) or *dsx* (lane 10) in XX flies causes a switch to the male expression pattern.

As a first step towards determining any potential SXL-mediated regulation, we analyzed the tissue-specificity of the sex-specific transcripts for five of the remaining six candidates. The sixth candidate, *Act5C*, had several homologs that were highly conserved at the nucleotide level, raising the possibility that several individual genes likely contributed to the pattern of transcripts observed in Fig. 2. Thus, this candidate was not characterized further at this time. The expression patterns of the remaining five candidates were analyzed in the progeny of *tudor* (*tud^1^/Df*) flies, which lack a germline, allowing the sex-specific transcripts to be sorted based on somatic or germline origin. Three of the five candidates showed consistent, reproducible results. The shorter male-specific transcripts found in two of the candidates, CG11737 and *blown-fuse (blow)*, remained in the progeny of *tudor* (*tud^1^/Df*) flies (Figure 4A, panels 1 and 2, lane 3 versus lane 1) indicating that the transcripts are somatic in origin. The third candidate, *Rm62*, exhibited the opposite effect. The shorter female-specific transcript was not present in the progeny of *tudor* (*tud^1^/Df*) flies (Figure 4A, panel 3, lane 4 versus lane 2), indicating that this transcript is germline specific.

Although the function of CG11737 is not known, the functions of *blow* and *Rm62*, were intriguing. *Blow* is implicated in somatic muscle development [32], and a male-specific somatic muscle had been previously described [33]. *Rm62* is an mRNA-binding protein with ATP-dependent helicase activity that has been implicated in alternative splicing [34], [35], making it a potent potential downstream target of SXL.

### Candidates CG11737 and *blow* are downstream of *dsx* in the soma

Expression of the sex-specific transcripts of CG11737 and *blow* in somatic tissue raised the possibility that they might be targets of SXL in the soma. Given that both of the sex-specific transcripts were seen in males and that SXL is not present in males, the most likely role for SXL would be the repression of these male-specific transcripts in females. To test this hypothesis, we examined the expression pattern of CG11737 and *blow* in female flies lacking somatic SXL (Figure 4B, lane 3). Loss of SXL in these flies caused the appearance of the male-specific transcript of both CG11737 and *blow*. This indicated that both CG11737 and *blow* are downstream of *Sxl* in the soma.

Although CG11737 and *blow* were downstream of *Sxl*, the observed effect could be due to indirect regulation through *tra* and *dsx*. Therefore, we tested the effects of loss of TRA and DSX^F^ on the expression of the male-specific transcript. For both CG11737 and *blow*, loss of *tra* (Figure 4C, lane 4) or loss of *dsx* (Figure 4C, lane 10) in females caused switching to a male expression pattern. Thus, the sex-specific expression patterns of CG11737 and *blow* are governed by genes in the somatic sex-determination pathway downstream of *Sxl*. Moreover, these findings emphasize that presence of an SXL-binding site is necessary but not sufficient for SXL-mediated regulation. We conclude that the two genes are indirectly regulated by SXL.

### *Rm62* is downstream of SXL in the germline

**Figure 5:**
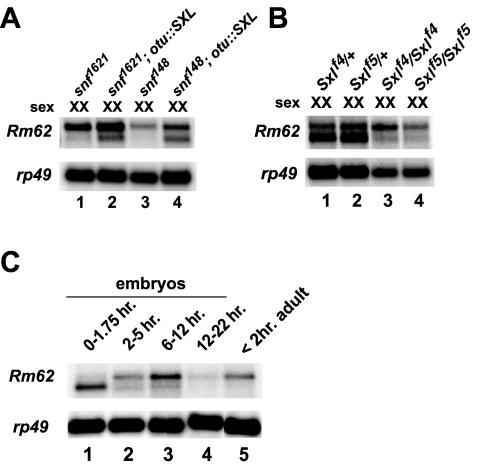
Germline SXL is necessary for expression of the female-specific *Rm62* transcript. A. XX flies homozygous for the *snf^1621^* or *snf^148^* alleles, which disrupt SXL function specifically in the germline, do not express the female-specific *Rm62* transcript (lanes 1 and 3). Expression of a *Sxl* cDNA in *snf* mutant backgrounds under the control of the *otu* promoter restores the synthesis of the female-specific *Rm62* transcript (lanes 2 and 4). B. XX flies homozygous for the *Sxl^f4^* or *Sxl^f5^* alleles show a loss of the female-specific *Rm62* transcript (lanes 3 and 4). C. The female-specific *Rm62* transcript is maternally deposited, and is present only in mature ovaries.

Since the female-specific transcript of *Rm62* is expressed in the germline, we tested whether SXL function in the germline was necessary for its production. First, we examined *Rm62* expression in the flies with mutations in the *sans fille (snf)* gene (*snf^1621^* and *snf^148^*) that disrupt *Sxl* function in the female germline. Female flies homozygous for either *snf* mutation did not express the female-specific *Rm62* transcript (Figure 5A, lanes 1 and 3), but introduction of an *Sxl* cDNA into these backgrounds restored expression of the transcript (lanes 2 and 4). Second, females homozygous for the *Sxl^f4^* and *Sxl^f5^* alleles also lacked the female-specific *Rm62* transcript (Figure 5B, lanes 3 and 4 versus 1 and 2). The combined results of these experiments demonstrate that SXL function in the germline is necessary for expression of the female-specific, shorter *Rm62* isoform either directly or indirectly.

Given that the female-specific *Rm62* transcript was produced in the germline, it was possible that it was maternally deposited. Examination of the *Rm62* expression pattern in embryos showed that the female-specific *Rm62* transcript was specifically deposited into embryos (Figure 5C, lane 1) but was replaced by the non-sex-specific transcript after the maternal to zygotic transition (Figure 5C, lanes 2–4). We conclude that *Rm62* is downstream of SXL in the female germline and is maternally deposited.

Thus, our genome-wide search fulfilled its main purpose - to identify SXL targets near splice sites that showed evidence of alternative splicing, to identify all of the previously known targets, and to identify a novel target *e(r)* and a potential target *Rm62*. Only a subset of SXL-binding sites are regulated by SXL *in vivo*. The *e(r)* and *Rm62* transcripts provide important downstream handles to study the mysterious role of SXL in the female germline.

## Discussion

The genome-wide screen presented here, combining a computational search, biological constraints, and molecular genetic analysis, identified both the previously known targets and a novel target of SXL. Identification of transcripts that appear downstream of SXL in the female germline is an important step toward understanding the role of SXL in the female germline.

Although previously known targets of SXL (*tra* and *msl2*) have exclusively sex-specific functions, it is not unreasonable to expect that certain targets could have both non-sex-specific and sex-specific functions at different times or in different tissues during development. These targets could have easily escaped previous genetic screens that identified the known components of the sex-determination pathway based on sex-specific phenotypes. In fact, the germline-specific *Sxl* target *e(r)*, which was identified in this screen, is essential in both sexes during embryogenesis and is regulated by *Sxl* in the female germline later during development [31]. Similarly, whereas certain mutations in the *Drosophila* PTB and the class VI unconventional myosin 95F (*jaguar*) are lethal, others specifically affect spermatogenesis, resulting in a male-sterile phenotype indicating that a gene can have both non-sex-specific and sex-specific function and/or regulation [36], [37], [38]. Therefore, we believe that additional *Sxl* targets that contribute to sexual dimorphism but do not solely have sex-specific functions remain to be identified. Among the potential new candidates that have SXL-binding sites in relevant biological contexts (Figure 1B) and that show sexually dimorphic expression patterns (Figure 2), *Rm62* is a potential target in the female germline, and the others (*blow*, CG3630, CG6422, and CG11737) are indirectly regulated by SXL via *dsx* in the soma. *blow* encodes a protein necessary for myoblast fusion and proper mesoderm development during embryogenesis, and the remaining candidates CG3630, CG6422, and CG11737 have no known function or previously recognized protein domain structure, although CG6422 is a putative member of the YT521-B-like family, which has been shown to modulate splice site selection *in vivo* [32], [39], [40]. *Rm62*, which is an ATP-dependent RNA helicase that contains a DEAD-box domain and an RRM-type RNA-binding motif [41], [42], is inferred to be involved in the regulation of alternative splicing [35] and interacts with components of the RNAi machinery [43]. Given that the computational screen identified the three known targets of SXL as well as a novel target of SXL, characterization of *Rm62* using molecular genetics should provide important new insights into the function and regulation of both previously characterized and uncharacterized genes, the mechanisms of action of SXL, and the basis for sexual dimorphism. Known molecular differences in the *Rm62* transcripts represent both alternative splicing and polyadenylation variants. Future studies should address whether *Rm62* is a direct or indirect target of SXL, and what the molecular basis for the sex-specific *Rm62* transcript is.

Independent biological information from one or more genome-wide analyses may also be integrated to further refine the list of candidates. For example, SXL may collaborate with cofactors for increased specificity, as documented for the repression of *msl2* translation in females by SXL and its cofactor UNR [44], [45], and for sex-specific splicing regulation of the *dsx* and *fruitless* transcripts by TRA and its cofactor TRA2 [10]. Second, conservation of short degenerate binding sites for RNA-binding proteins may be revealed by cross-species sequence comparison as has been done for *C. elegans* [46]. Third, direct *in vivo* RNA binding can be revealed by immunoprecipitation coupled with microarray analysis as has been done in *Drosophila* [47]. Thus, incorporation of additional biological constraints should help overcome limitations unique to any given approach and reduce the number of candidates for detailed characterization. Incorporation of such constraints could also allow searches for clusters of short uridine-rich sequences, which occur frequently and were ignored in the present study.

We note that recent experiments employing tiling arrays argue for extensive transcription in the *Drosophila* genome [48], implying that a subset of the putative SXL binding sites in regions annotated as intergenic could have a role in post-transcriptional regulation of non-coding transcripts.

In the future, the computational search presented here could benefit from improvements in the following areas: availability of full-length ESTs; improved annotations of gene structure, especially at exon/intron junctions; identification of low-abundance alternative transcripts; incorporation of quantitative information with respect to the frequency of alternatively spliced isoforms; and improvement in the speed of the algorithm by using indexing techniques and relational databases [49].

The majority of RNA-binding proteins, including splicing regulators, tend to have short, degenerate binding sites that occur frequently throughout the genome. Therefore, identification of biologically relevant binding sites is one of the most important challenges in the area of gene regulation. The most important strength of the analysis presented here is that the SXL binding site, although frequent throughout the genome, has biological consequences only when present in specific contexts such as in the proximity of alternative splice or polyadenylation sites. Moreover, since not all SXL-binding sites are regulated by SXL, a co-factor may collaborate with SXL to specify those that are regulated. Our computational method is readily adaptable to any RNA-binding protein for which a consensus binding sequence is known but the targets are unknown [50], and should provide a powerful tool in the search for target pre-mRNAs.

In conclusion, this approach has identified a novel target of SXL, has provided an additional potential target, and can be extended to numerous other RNA-binding proteins for which binding sites have been identified.

## Materials and Methods

### Databases and indexing

We downloaded the databases for the *Drosophila* genome (na_geno.dros.RELEASE2.Z) from the Genome Annotation Database of Drosophila Release 2 (GADFLY) (http://www.fruitfly.org/sequence/dlMfasta.shtml) and the expressed sequence tag (EST) database (na_EST.dros.Z) (http://www.fruitfly.org/sequence/dlcDNA.shtml) from the Berkeley Drosophila Genome Project (BDGP). Both the genomic and EST databases were converted, using the formatdb command, from a fasta format to a format usable by BLAST using the NCBI toolbox (ftp://ftp.ncbi.nih.gov/toolbox/). In addition, both databases were indexed to fetch sequences faster for intermediate steps during analysis.

### Generation of the weight matrix for the SXL binding site

The SXL weight matrix for the search was created from twenty-six sequences selected by selection-amplification from a random RNA library [20]. First, the sequences were arranged into an alignment matrix, which defined the number of times each nucleotide was found at a specific position within the alignment. The alignment matrix was then converted into a weight matrix using the formula:
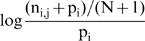
where N is the total number of sequences, p_i_ is the a priori probability of nucleotide i, and n_i,j_ is the number of times nucleotide i appears at position j (for detailed description see [24]).

### Sequence search

To identify SXL-binding sites, overlapping windows of 16 nucleotides were scored using the weight matrix (Table S1), and strings that scored higher than the cut-off value of 5.1 were labeled as potential high-affinity binding sites. The highest possible score, which can be obtained by adding the highest value in each row of Table S1, is 7.88 for the SXL matrix.

### Biological filters

To determine if the identified binding site was near a gene, 6 kb of genomic DNA (3 kb on each side) was used to BLAST against the EST database using blastn. The blast results were used to align the ESTs against the genome using ClustalW. Each alignment was automatically screened in two ways. First, only alignments that had both putative exons and introns within 100 bases of the binding site were retained. Second, the putative exon and intron junctions were examined, and those that matched at least partially the splice-site consensus signals received high priority. Splice sites were identified by searching the genome with weight matrices created using the consensus 5′ and 3′ splice site signals [24], [25]. To search for SXL-binding sites in the EST database, every EST containing a binding site was aligned with other ESTs from the same CLOT (a group of homologous ESTs as defined by BDGP); some CLOTs contained too many EST sequences to be aligned, and were skipped. For each binding site the alignments were converted into a post-script file and examined manually to ensure that they met the above criteria.

All of the programs used here were written in Perl (http://www.perl.org/). The entire code and instructions for its use are available upon request.

### cDNAs for analysis

ESTs, shown in parenthesis, for the following candidates were purchased from the Research Genetics Inc., CA: CG3630 (HL02887), *bancal* (LD15857), CG6422 (LD12853), *Bap60* (LD19076), *ImpL3* (LP10507), *inx7* (GH21056), *fus* (GH20047), *pUf68* (GH10982), CG11737 (LP01982), *Moe* (GH06344), *Rala* (SD01661), *Ric* (GH14071), CG8370 (LD46954), *Top2* (GH09845), *katanin-60* (SD02251), *vap* (LP02818), *Rm62* (LD17967), *Frq2* (LP01723), CG2967 (GH19107), CG5455 (GH11517), *Cyp28a5* (GH10483), *Act5c* (GH04613), *blow* (LP06243), *RpL36* (LP12131), *aralar1* (GH01348), *Dhc16F* (LP05023), and *e(r)* (LD36385). ESTs HL02887, LD15857, LD12853, LD19076, and LD17967 had been cloned into the pBluescript SK+ vector, and ESTs LP10507, GH21056, GH20047, GH10982, LP01982, GH06344, SD01661, GH14071, LD46954, GH09845, SD02251, LP02818, LP01723, GH19107, GH11517, GH10483, GH04613, LP06243, LP12131, GH01348, LP05023, and LD36385 had been cloned into the pOT2 vector. Templates for Northern blot probes were generated by PCR from the pBluescript SK+ ESTs using the T7 primer (5′ GTAATACGACTCACTATAGGG 3′) and the T3 primer (5′ AATTAACCCTCACTAAAGGG 3′, and from the pOT2 ESTs using the T7 primer and the pm001 primer (5′ CGTTAGAACGCGGCTACAAT 3′).

### poly(A)^+^ RNA extraction

Total RNA was isolated using TRI reagent (Sigma-Aldrich, MO). Poly(A)^+^ RNA was isolated using the PolyATtract mRNA isolation system (Promega, WI).

### Northern analysis

For each lane, approximately 0.5–1.0 µg of poly(A)^+^ RNA was separated by electrophoresis on a 1% agarose gel containing formaldehyde. RNA was transferred to a Duralose-UV membrane (Stratagene, CA), hybridized with ^32^P labeled probe at 42°C overnight, washed extensively, and imaged on a Molecular Dynamic Phosphorimager. Additional details for various genotypes (*tra*, *Sxl*, *dsx*, and *tud*) can be found in [31], [36]

## Supporting Information

Table S1 A weight matrix for the SXL binding site. Underlined positions reflect preferred residues.
(0.44 MB TIF)
