# Supplementary figures and images for: Genome-Wide Identification of Alternatively Spliced mRNA Targets of Specific RNA-Binding Proteins
Source: PLoS One. 2007 Jun 13;2(6):e520. doi: 10.1371/journal.pone.0000520 (PMC1885218; doi:10.1371/journal.pone.0000520)

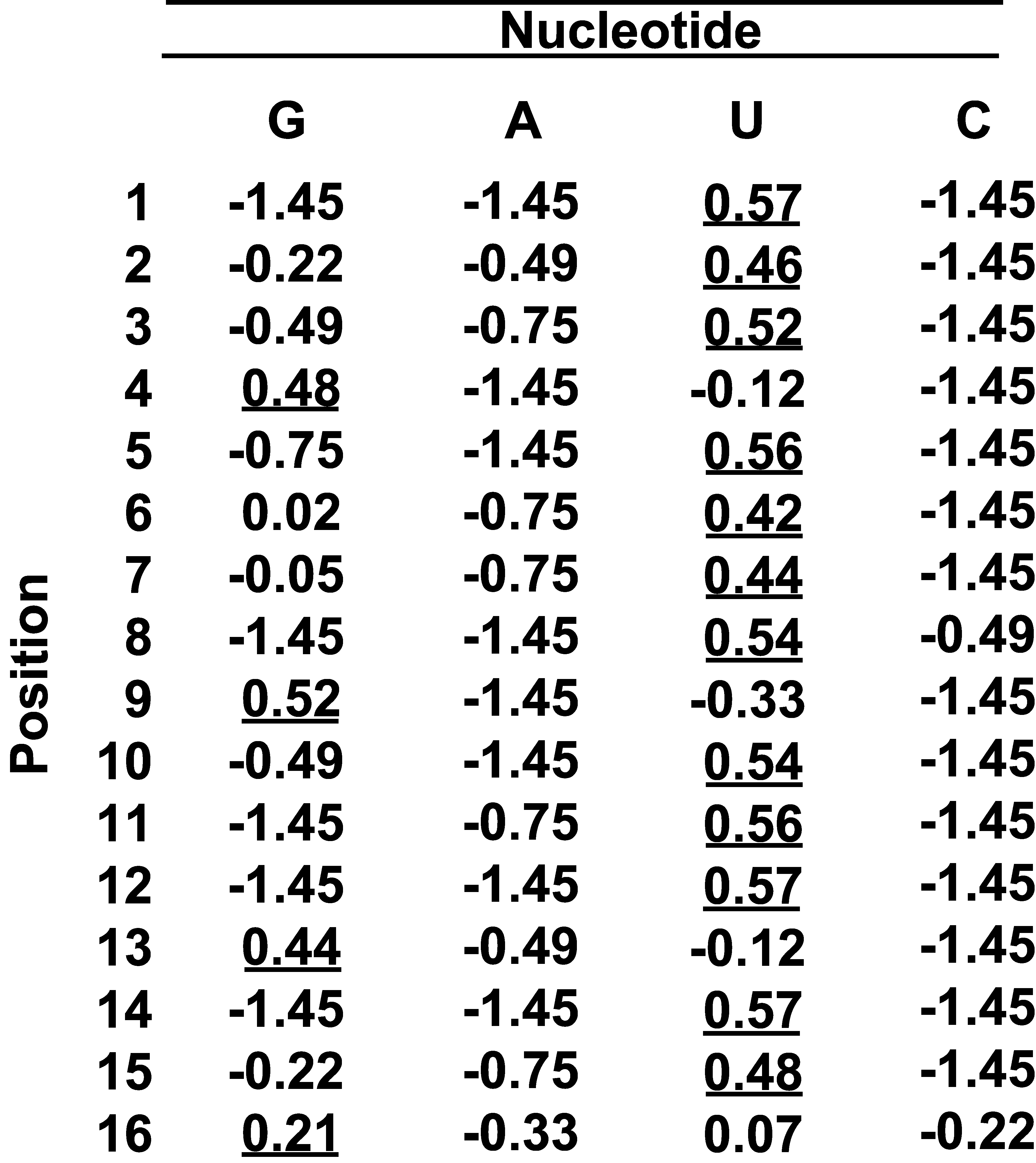

Supplement: Table S1 — A weight matrix for the SXL binding site. Underlined positions reflect preferred residues. (0.44 MB TIF) [file pone.0000520.s001.tif]
